# Rural-Urban Trends in Congenital Heart Disease-Related Mortality in the United States, 1999 to 2019

**DOI:** 10.1016/j.jacadv.2022.100030

**Published:** 2022-05-18

**Authors:** Abdul Mannan Khan Minhas, Rachel A. Wyand, Robert W. Ariss, Salik Nazir, Vardhmaan Jain, Sadeer G. Al-Kindi, Michael D. Shapiro, William Campbell, Laurence Sperling, Salim S. Virani

Despite continuously declining congenital heart disease-related mortality in urban counties from 1999 to 2019, rural counties had persistently higher mortality, which did not decline significantly from 2009 to 2019.

Congenital heart disease (CHD) affects about 1 in 100 live births per year in the United States (US). Advances in surgical and medical care have extended life expectancy over the past 2 decades in patients with CHD. Cardiovascular mortality, risk factors, and barriers to health care have been reported to be higher in rural areas in the U.S.^1^^,^^2^ Considering the complex, multidisciplinary care and increasing life expectancy among those living with CHD, patients residing in rural areas may experience disparate outcomes compared to those living in urban areas. However, there is a paucity of data examining rural-urban disparities in CHD-related mortality. To further investigate this issue, we report trends in CHD-related mortality by rural-urban county classification over 2 decades.

Deaths occurring in the U.S. from 1999 to 2019 with CHD as an underlying or contributing cause were extracted from the Centers for Disease Control and Prevention Wide-Ranging OnLine Data for Epidemiologic Research database. CHD-related deaths of all ages were identified with International Classification of Diseases-10th Revision Clinical Modification codes Q20 to Q26 as previously reported.^3^ The study was exempt from institutional review board approval because the data are publicly available and anonymized. Using the National Center for Health Statistics Urban-Rural Classification Scheme, the study population was divided into urban (population ≥50,000) and rural (population <50,000) counties per the 2013 U.S. census classification. Age-adjusted mortality rates (AAMRs) per 1,000,000 people and associated annual percent change (APC) with 95% confidence intervals (CIs) were calculated. Comparisons between urban and rural average AAMR were tested using z test statistic, with *P* < 0.05 considered statistically significant.

**Figure 1:**
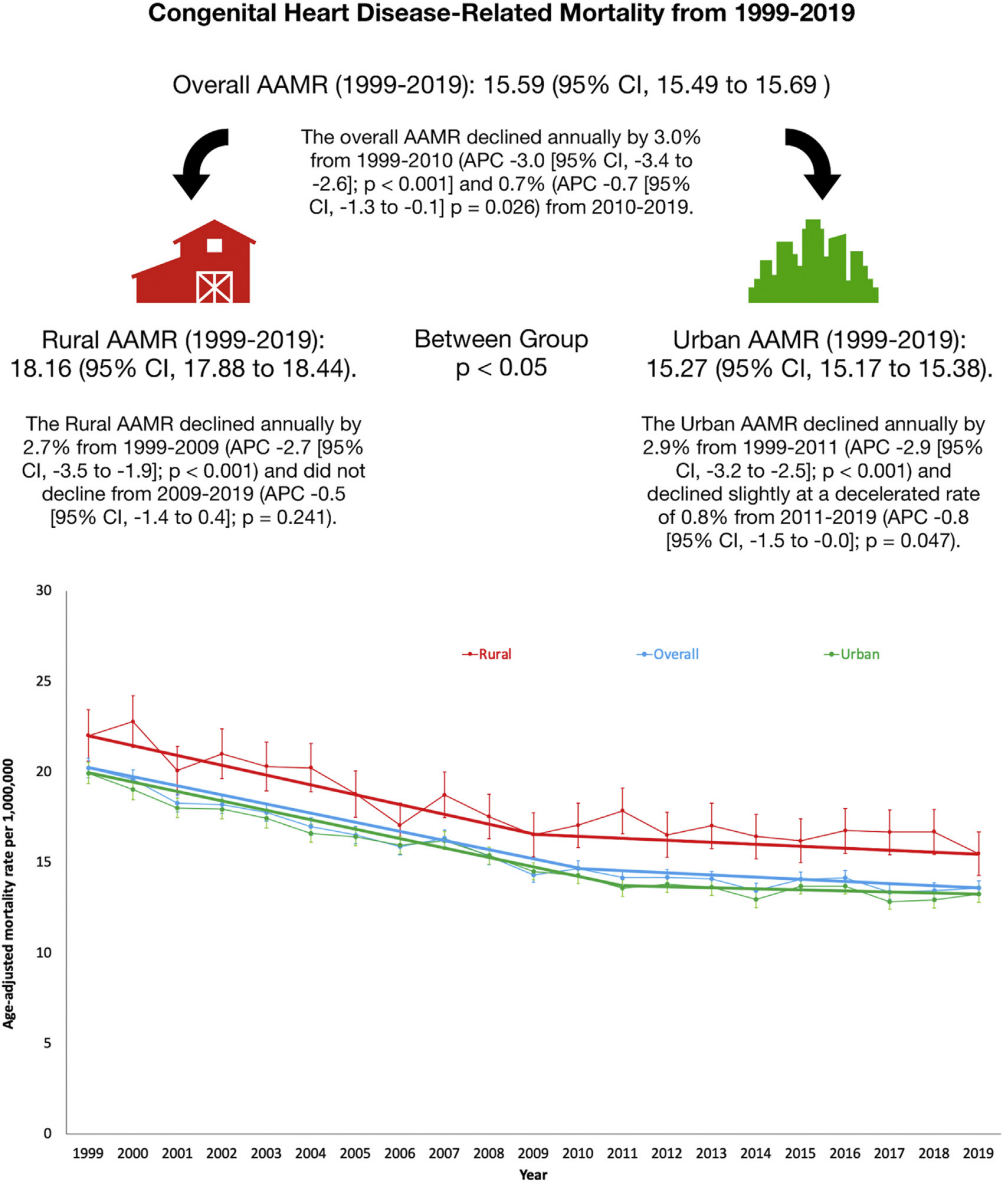
**Trends in Congenital Heart Disease Mortality by Rural-Urban Designation in the United States, 1999 to 2019** Error bars indicate 95% confidence interval of the age-adjusted mortality rates.

Between 1999 and 2019, a total of 98,282 CHD-related deaths occurred in the study population. Of those deaths, 46,288 (47.10%) were during an age <1 year, 5,832 (5.93%) at 1 to 5 years, 4,490 (4.57%) at 6 to 17 years, and 41,667 (42.40%) at ≥18 years of age. The AAMR for CHD-related deaths was 20.21 in 1999 and 13.58 in 2019. The overall AAMR declined from 1999 to 2010 (APC −3.0; *P* < 0.001) and from 2010 to 2019 (APC −0.7; *P* = 0.026). The overall AAMR in rural counties was significantly higher than that in urban counties (rural: 18.16 [95% CI, 17.88-18.44] vs urban: 15.27 [95% CI, 15.17-15.38]; *P* < 0.05). The AAMR in rural counties was 22.0 in 1999 and 15.5 in 2019. Urban counties had an AAMR of 19.9 in 1999 and 13.2 in 2019. In urban counties, AAMR declined from 1999 to 2011 (APC −2.9; *P* < 0.001). From 2011 to 2019, a slight, decelerated decline was seen in AAMR (APC −0.8; *P* = 0.047). Following an initial decline in AAMR from 1999 to 2009 (APC −2.7; *P* < 0.001), the AAMR in rural areas did not decline significantly from 2009 to 2019 (APC −0.5; *P* = 0.241) (Figure 1).

In this study, rural counties had consistently higher CHD mortality than urban counties, and the overall CHD-related mortality within rural counties did not decline significantly from 2009 to 2019. Conversely, urban counties experienced continuously declining CHD mortality throughout the study period although the decline decelerated from 2011 to 2019. There are several plausible explanations for these findings. First, rural populations have less access to specialized CHD care as most specialty centers are located in major cities.^1^ Adults with CHD who receive care in specialized centers are more likely to live in urban areas and have a significant reduction in mortality compared to those receiving care in nonreferral center areas.^4^ In a prior study, maternal residence in closer proximity to a top-50-ranked pediatric cardiac care center was associated with lower mortality risk in infants with CHD,^5^ demonstrating the barriers in access to care for rural populations living farther from these centers. Second, rural populations have a greater burden of socioeconomic factors affecting their care including experiencing poverty, unemployment, and lack of transportation.^1^ These factors may contribute to higher CHD mortality seen within this study for rural populations.

In conclusion, despite declining CHD-related mortality in urban counties, rural counties had persistently higher mortality which did not decline significantly from 2009 to 2019. These trends require initiatives to ensure improved access to specialized CHD health care and address socioeconomic barriers experienced by rural populations.
